# The effect of sample storage on the Peroxidation of Leukocytes Index Ratio (PLIR) measure

**DOI:** 10.1038/srep06539

**Published:** 2014-10-07

**Authors:** Ilaria Peluso, Husseen Manafikhi, Fabio Altieri, Christian Zanza, Maura Palmery

**Affiliations:** 1 Department of Physiology and Pharmacology “V. Erspamer”, “Sapienza” University of Rome, Italy; 2 Department of Biochemical Sciences “Alessandro Rossi Fanelli”, “Sapienza” University of Rome, Italy

## Abstract

Delays in processing are frequent because of problems associated with transporting the samples to the laboratory. Thus, we aimed to evaluate the effect of sample storage on the Peroxidation of Leukocytes Index Ratio (PLIR). Differences between PLIR values of lymphocytes (PLIR-L), monocytes (PLIR-M) and granulocytes (PLIR-G) were observed in fresh samples. Sample storage affected the evaluation of PLIR. In particular, PLIR-L was lower in stored samples compared to fresh samples. In conclusion, our results suggest that fresh samples are recommended for assessing the PLIR.

The oxidation products of lipids, DNA and proteins and/or the enzymatic and non enzymatic antioxidants are usually measured in order to evaluate the oxidative stress, associated with many pathological and sub-clinical conditions^1^. However, the major bias of the total antioxidant capacity methods is that they are strongly influenced by the presence of products of catabolism, such as bilirubin and uric acid^2^,^3^. Furthermore, the FRAP method (Ferric Reducing Antioxidant Potential) matches the antioxidant capacity to the reducing ability^3^. It is well known that reduced iron is critical in the onset of oxidative stress due to the Fenton reaction, that generates the hydroxyl radical initiator of lipid peroxidation^1^. On the other hand, the role of uric acid and bilirubin in the prognosis of oxidative stress-related diseases is still controversial^4^,^5^.

From the computational analysis of markers of oxidation (plasma malondialdehyde, oxidized glutathione and urinary isoprostanes) and antioxidants (reduced glutathione, tocopherol and plasma antioxidant capacity) an index of global oxidative stress (OXY-SCORE) has been proposed^6^. However, this type of approach pictures only the redox state without considering the important role of free radicals in the innate response (NADPH-oxidase, myeloperoxidase) and in the resistance to infection, that declines during aging^7^.

The Peroxidation of Leukocytes Index Ratio (PLIR)^8^ is a test that measures both the resistance of leukocytes to an exogenous oxidative stress and the leukocytes functional capacity of oxidative burst upon activation. Furthermore, unlike the intracellular probes, PLIR is not affected by the interference of substrates of the multidrug resistance proteins^9^. Therefore, PLIR could be a flexible method to study the redox balance in both preclinical and clinical conditions, as well as to evaluate the effects of pharmacological or nutritional interventions. However, delays in processing are frequent because of problems associated with transporting the samples to the laboratory.

Therefore, we aimed to evaluate the effect of sample storage on the PLIR method.

## Results and Discussion

**Figure 1:**
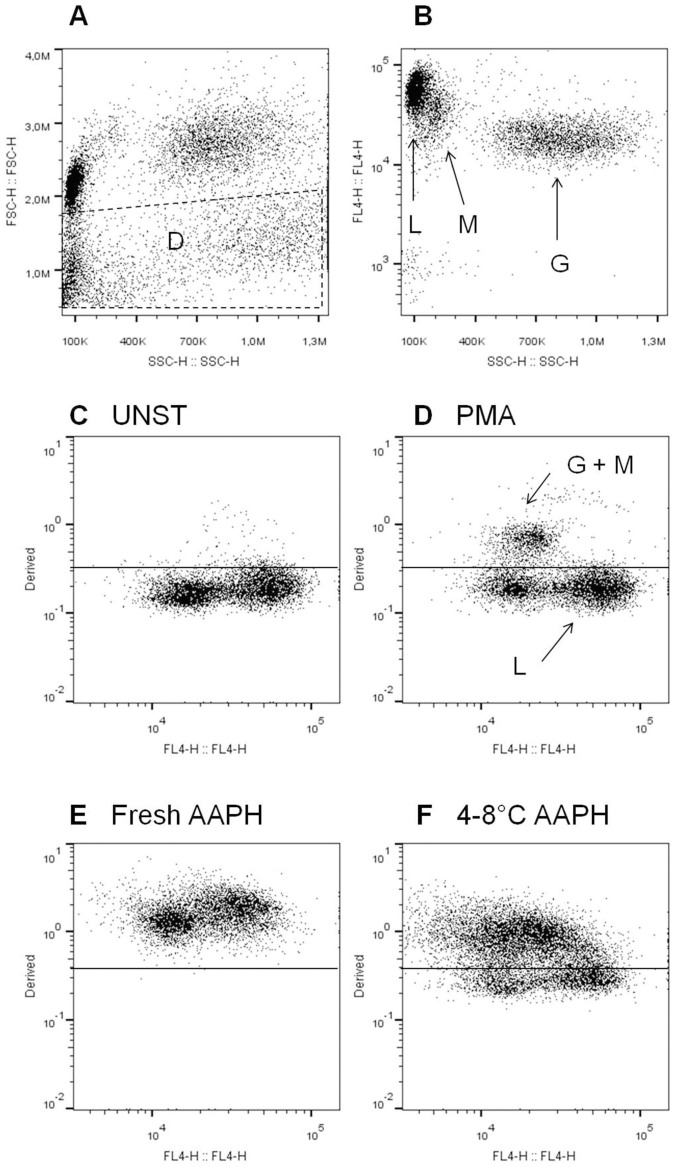
Typical dot plots FSC vs. SSC (A) and FL4 (CD45-APC) vs. SSC (B); L: lymphocytes, M: monocytes, G: granulocytes, D: dead cells and/or debris. Typical dot plots Derived (FL1/FL2) vs. FL4 of cells un-stimulated (UNST) (C) or treated with PMA (1 μg/ml) (D) or AAPH (10 mM) (E and F) for 30 min.

After the exclusion of dead cells and/or debris (D) on forward (FSC) versus (vs.) side scatter (SSC) plots, lymphocytes (L), monocytes (M) and granulocytes (G) were selected by CD45-APC vs. SSC plots (Fig. 1).

The fluorescent probe 4,4-difluoro-5-(4-phenyl-1, 3-butadienyl)-4-bora-3a, 4a-diaza-s-indacene-3-undecanoic acid (C11-BODIPY), used in the PLIR method, modifies its fluorescence from red (FL2) to green (FL1)^8^,^10^ as a result of oxidation. We observed both the expected increase in FL1 and decrease in FL2 upon oxidation, contrarily to the unexpected increase of C11-BODIPY fluorescence at 600 nm observed by measuring the progression of neutrophil membrane oxidation upon activation by monitoring the decay of red fluorescence^11^.

The oxidized/reduced fluorescence ratio of C11-BODIPY has been used in order to normalize for cell incorporation of the probe into membrane^8^. We derived the RATIO (FL1/FL2) by FlowJo software. Treatment with 2,2′-azobis(2-methylpropionamidine) dihydrochloride (AAPH) or phorbol 12-myristate 13-acetate (PMA) treatment increased the RATIO of fluorescence, but in a different manner, showing that oxidative burst induced reactive oxygen species (ROS) production only in activated cells, while all cells were sensitive to exogenous ROS injury (Fig. 1). The vitamin E analogue 6-hydroxy-2,5,7,8-tetramethylchroman-2-carboxylic acid (Trolox), did not affect baseline level of oxidation and inhibited the peroxidation of C11-BODIPY in leukocytes exposed to AAPH free radicals generating system. The AAPH-induced oxidation was greater in fresh samples than in 4–8°C stored samples (Fig. 1). From the gated populations on CD45-APC vs. SSC plots (Fig. 1), we calculated the PLIR-L, PLIR-M and PLIR-G as previously described^8^.

Statistical analysis, carried out with Friedman Repeated Measures Analysis of Variance (RM ANOVA) on Ranks, revealed a normal distribution for L, M and G populations (Normality Test Shapiro-Wilk passed: L: p = 0,308; M: p = 0,386; G: p = 0,326).

One Way RM ANOVA, with type of sample as within-subjects factor, revealed that the differences in the mean values among the sample groups were greater than would be expected by chance for L (P = 0.002; Power of performed test with alpha = 0.050: 0.916) and M (p = 0,004; Power of performed test with alpha = 0.050: 0.839). To isolate the group that differs from the others we used the Bonferroni post-hoc analysis (All Pairwise Multiple Comparison Procedure).

**Figure 2:**
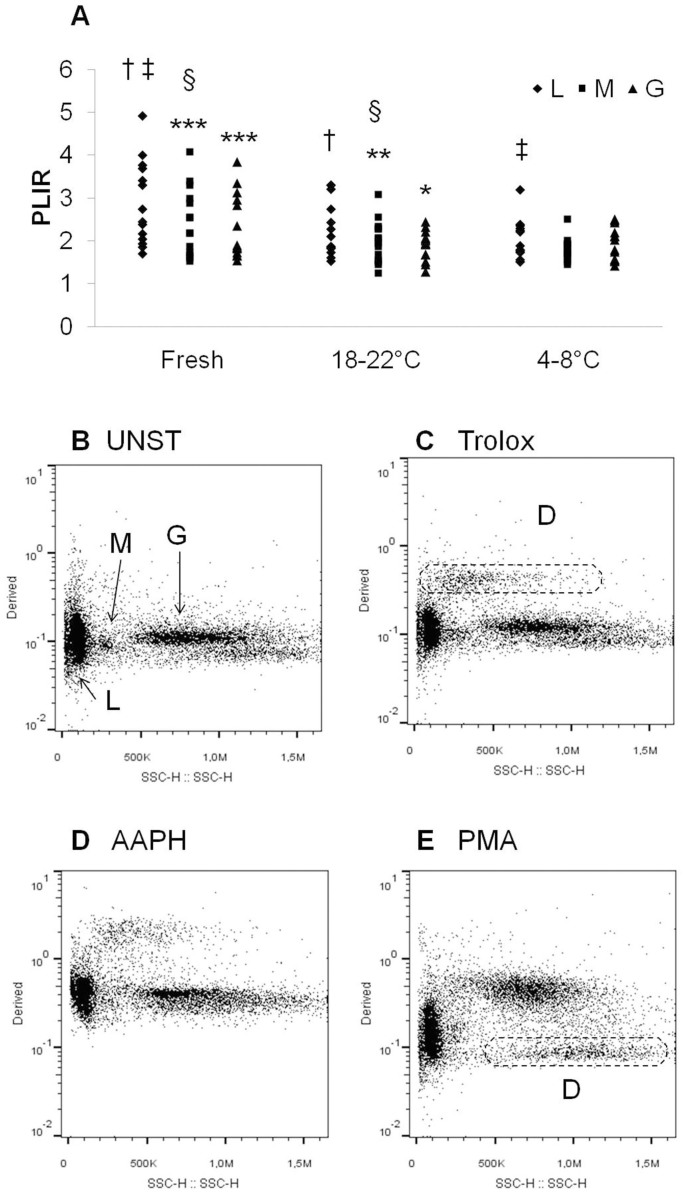
Raw data of PLIR values of lymphocytes (L), monocytes (M) and granulocytes (G), measured in fresh samples or samples stored at 18–22°C or 4–8°C for 24 h (A). Comparisons for factor sample within L: †p < 0.05, ‡p < 0.01; within M: §p < 0.01. Comparisons for factor cell within sample: L versus G or M **p < 0.01, ***p < 0.001. Typical dot plots Derived (FL1/FL2) versus SSC of L, M, and G from samples stored at 4–8°C for 24 h, un-stimulated (UNST) (B) or treated with Trolox (10 μM) (C), AAPH (10 mM) (D) or PMA (1 μg/ml) (E). D: dead cells and/or debris.

Decreases in PLIR-L (fresh vs. 4–8°C: p = 0.002; fresh vs. 18–22°C: p = 0.026; power of performed test with alpha = 0.050: 0.916) and PLIR-M (fresh vs. 4–8°C: p = 0.004; power of performed test with alpha = 0.050: 0.839) were observed in the stored samples compared to fresh samples (Fig. 2).

In order to investigate the possible interaction between sample storage and cell type, we performed two way RM ANOVA, with sample or cell type as within-subjects factors (Equal Variance Test passed: p = 0.826; power of performed test with alpha = 0.05 for sample: 0.875, for cell: 0.999, for sample × cell: 0.645). There was a significant interaction between sample and cell (P = 0.016). In order to isolate differences between groups, the Bonferroni post-hoc analysis was used.

In fresh samples PLIR-L was significantly different compared to PLIR-M (p < 0.001) and to PLIR-G (p < 0.001) (Fig. 2).

The differences between the PLIR values of different populations were attenuated or lost after storage at 18–22°C (PLIR-L vs. PLIR-G p = 0.002; PLIR-L vs. PLIR-M p = 0.03) or 4–8°C, respectively (Fig. 2).

These results could be due to the high percentage of D events observed in the 4–8°C stored samples (Fig. 2). In particular, a population, resembling the apoptotic eosinophils described by Sandström *et al.*^12^, showed neither the increase in the RATIO of fluorescence nor the reduction of SSC after PMA-activation (Fig. 2). On the other hand, after AAPH treatment an increase in the oxidation was seen in D events (Fig. 2). Therefore, despite D events had been excluded from the PLIR analysis, they could scavenge cells from free radicals generated by AAPH and their presence in the reaction tube could interfere with the oxidation of the other cells.

On the other hand, the AAPH-induced oxidation in the 4–8°C stored samples could be due to changes in the antioxidant capacity after storage. In particular, it has been reported that the quality of stored blood can be deteriorated by haemolysis caused by free radicals. Markers of haemolysis and free radical metabolism have been previously examined in samples with high (HL) and low (LL) leukocyte count^13^. Authors observed an increase in the antioxidant capacity in blood samples stored at 4°C, which was accompanied by haemolysis^13^. In post-storage samples, the increase of the lipoperoxidation marker malondialdehyde was significantly higher in HL samples, suggesting that leukocytes participate in free radical production in stored blood. Besides, it is known that bilirubin, the product of haemoglobin catabolism, increases the antioxidant capacity^2^,^3^,^4^, whereas iron is involved in the generation of the lipid peroxidation^1^. Therefore, haemolysis during storage could affect the PLIR evaluation. These considerations could also explain the Trolox-induced increases in the percentage of D events in some of the PMA-activated samples stored at 4–8°C (Fig. 2). This effect was probably due to the iron reducing activity^3^, which could increase the Fenton reaction subsequent to hydrogen peroxide production induced by oxidative burst. On the other hand, a less pronounced protection on AAPH-induced oxidation has been observed, probably due to the loss of the known synergisms between antioxidants^2^. In support of this hypothesis, plasma antioxidant capacity it is known to decrease with storage^2^.

Overall these results suggest that PLIR is sensitive to the concentration of plasma antioxidants, as well as to the products of catabolism and iron generated by haemolysis.

Interestingly, on fresh samples we found PLIR ranges (PLIR-L Range 1.70–4.92; PLIR-M Range 1.53–4.07; PLIR-G Range 1.54–3.83) larger than those previously reported (PLIR-L Range 0.69–1.64; PLIR-M Range 0.84–1.26; PLIR-N Range 0.84–1.15)^8^. It is possible that the filters of the flow cytometer Accuri BD (FL1 533/30 nm; FL2 585/40 nm) are better centred on the two fluorescence of C11-BODIPY, compared to those of the flow cytometer Epics XL Coulter (FL1 525/20 nm; FL2 575/25 nm; FL3 620/15 nm), thus increasing the sensitivity^14^,^15^. Further studies are needed to evaluate the best filter tool to measure PLIR.

In conclusion, we have observed a significant difference between PLIR of different leukocytes subset on fresh samples. Such differences were attenuated after storage of the samples. Therefore, our results suggest that fresh samples are recommended for assessing the PLIR.

## Methods

This work can be considered an observational and/or a quality control/improvement project. Furthermore, taking a single blood sample from a healthy volunteer would generally only present a minimal risk, and might therefore be regarded as acceptable. Therefore no Regional Ethics Committee approval was required^16^, but all procedures involving human subjects complied with the Declaration of Helsinki as revised in 2000. Written informed consent was obtained from all the participants in accordance with Italian law (law n° 196/2003; Ministry of Health Circular Letter GU n° 76/2008).

Venous peripheral blood, from 14 healthy volunteers (10 men and 4 women, aged between 26 and 35 years), was collected after an overnight fasting. PLIR has been evaluated immediately after the collection and after 24 hours of storage at 4–8°C or at 18–22°C. After red blood cells' lysis and staining with C11-BODIPY (cod. D3861, lot. 1381315, Invitrogen Molecular Probes, final concentration 1 μM), leukocytes were treated as previously described^8^ with PMA (cod. P1585, lot. SLBC7913V, Sigma, final concentration 1 μg/ml), AAPH (cod. 440914, lot. MKBL5129V, Sigma-ALDRICH, final concentration 10 mM), Trolox (cod. 238813, lot. BCBM7406VP, Sigma-ALDRICH, final concentration 10 μM), PMA 1 μg/ml + Trolox 10 μM or AAPH 10 mM + Trolox 10 μM. After 30 min at 37°C cells were stored in ice, to stop reaction, and rapidly analyzed on an Accuri C6 BD cytometer. Cell surface staining of CD45 was performed with the anti-CD45-APC (clone HI30, isotype IgG1, κ, cod. 555485, lot. 3291614, BD Pharmingen).

Fluidic calibration of the Accuri C6 BD cytometer was performed before each experiment, as suggested by the manufacturer. Threshold was set at the FS parameter and samples were acquired until 10000 events were collected in the gate of CD45 positive cells.

Data acquired on the Accuri C6 was exported in FCS format and analyzed by FlowJo software to calculate the fluorescence ratio (FL1/FL2) as derived parameter. The PLIR was calculated, as previously described^8^, from the formula: 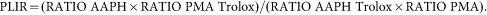


All statistical evaluations were performed using the SigmaStat and Sigmaplot software (Jandel Scientific, Inc.).
